# Neural Correlates of Ongoing Conscious Experience: Both Task-Unrelatedness and Stimulus-Independence Are Related to Default Network Activity

**DOI:** 10.1371/journal.pone.0016997

**Published:** 2011-02-14

**Authors:** David Stawarczyk, Steve Majerus, Pierre Maquet, Arnaud D'Argembeau

**Affiliations:** 1 Department of Cognitive Sciences, University of Liège, Liège, Belgium; 2 Cyclotron Research Centre, University of Liège, Liège, Belgium; University College London, United Kingdom

## Abstract

The default mode network (DMN) is a set of brain regions that consistently shows higher activity at rest compared to tasks requiring sustained focused attention toward externally presented stimuli. The cognitive processes that the DMN possibly underlies remain a matter of debate. It has alternately been proposed that DMN activity reflects unfocused attention toward external stimuli or the occurrence of internally generated thoughts. The present study aimed at clarifying this issue by investigating the neural correlates of the various kinds of conscious experiences that can occur during task performance. Four classes of conscious experiences (i.e., being fully focused on the task, distractions by irrelevant sensations/perceptions, interfering thoughts related to the appraisal of the task, and mind-wandering) that varied along two dimensions (“task-relatedness” and “stimulus-dependency”) were sampled using thought-probes while the participants performed a go/no-go task. Analyses performed on the intervals preceding each probe according to the reported subjective experience revealed that both dimensions are relevant to explain activity in several regions of the DMN, namely the medial prefrontal cortex, posterior cingulate cortex/precuneus, and posterior inferior parietal lobe. Notably, an additive effect of the two dimensions was demonstrated for midline DMN regions. On the other hand, lateral temporal regions (also part of the DMN) were specifically related to stimulus-independent reports. These results suggest that midline DMN regions underlie cognitive processes that are active during both internal thoughts and external unfocused attention. They also strengthen the view that the DMN can be fractionated into different subcomponents and reveal the necessity to consider both the stimulus-dependent and the task-related dimensions of conscious experiences when studying the possible functional roles of the DMN.

## Introduction

During the last decade, the default mode network (DMN) of the brain—a network of brain regions that includes the medial prefrontal cortex (MPFC), the posterior cingulate cortex (PCC)/restrosplenial cortex (Rsp), the medial and lateral temporal lobes, and the posterior inferior parietal lobes (pIPL)—has become the object of intensive focus and research in neuroscience [1], [2]. The DMN is particularly active during rest states (with a high degree of functional connectivity between its constituent regions) and shows reduced activity during a variety of demanding tasks in which sustained focused attention and the cognitive processing of externally presented stimuli is required [3], [4], [5], [6]. This decrease of activity is likely to reflect the suspension of cognitive processes that are active at rest [7], although the precise nature of these processes remains currently debated [8], [9], [10], [11].

Some authors have argued that higher DMN activity corresponds to a shift of perspective from current external information to internally generated cognitions [12], [13]. In keeping with this proposal, it has been found that subjective reports of task-unrelated thoughts during cognitive tasks (i.e., supposedly mind-wandering about past and future events) are related to increased activity in the DMN [7], [8], [11], [14], [15], [16]. Furthermore, DMN activity has been observed in a variety of tasks involving the generation of thoughts and images that are decoupled from the current external environment, such as in autobiographical memory retrieval, episodic future thinking, and social cognition [17], [18], [19], [20], [21], [22]. Finally, evidence from resting state functional connectivity studies indicates that the DMN is strongly negatively correlated with other brain regions usually engaged in the performance of cognitive tasks requiring sustained attention to external stimuli, including lateral prefrontal and parietal areas [23], [24].

The proposal that DMN activity corresponds to the occurrence of mind-wandering and internal cognitions is not consensual, however. Some authors have argued that DMN activity might support the general, unfocused monitoring of the external environment rather than internal thoughts [9], [25]. In line with this view, it has recently been shown that brief projections of task-unrelated visual stimuli during the maintenance phase of a working memory task result in increased activity in DMN regions [26]. Larger DMN activity has also been demonstrated in conditions where participants simply have to monitor the occurrence of external stimuli compared to conditions where these stimuli have to be maintained and manipulated in mind [27], [28]. Finally, it has also been argued [9] that the previously mentioned reports of task-unrelated thoughts do not necessarily reflect mind-wandering. They could as well represent the capture of attention by task-unrelated stimuli (e.g., external noises or hunger sensations, labeled here as external distractions [29], [30], [31]). The increased DMN activity that has been linked to task-unrelated reports might therefore reflect a state of unfocused attention in which salient interoceptive and exteroceptive perceptions that are irrelevant to the task at hand are nonetheless monitored.

These two views on the possible function of the DMN (i.e., mind-wandering and unfocused external attention) are not necessarily mutually exclusive. It is conceivable that activity in the DMN reflects a general state of unfocused attention in which both internal representations (i.e., thoughts and memories that are unrelated to the immediate environment) and task-unrelated external stimuli (i.e., interoceptive and exteroceptive information) are gathered and monitored [5], [32]. Another possibility would be that distinct regions of the DMN are involved in mind-wandering and unfocused external attention. Recent findings have indeed demonstrated that the DMN can be fractionated into different subcomponents [33], [34], [35], and some authors have proposed that, among the different regions of the DMN, the most rostral part of the MPFC is specifically related to unfocused attention towards external stimuli, while an adjacent but more caudal portion of MPFC would be specifically involved in considering one's own and others' mental states (i.e., mentalizing [9], [36]). Finally, it also remains to be determined whether the link between the DMN (or some of its constituent regions) and internal cognitions is specific to task-unrelated thoughts (e.g., mind-wandering about events that occurred in the past days or about what to do during the upcoming week-end) or whether it is also associated with thoughts related to the appraisal of the current task, such as thinking about the task length or about mistakes committed in past trials (i.e., task-related interferences [37], [38], [39]). In summary, DMN activity might reflect (1) the occurrence of thoughts that are both decoupled from stimuli present in the current environment and unrelated to the task currently being carried out (mind-wandering), (2) a state of unfocused attention towards external task-unrelated stimuli (external distractions), (3) attention towards self-generated internal thoughts, independently of whether or not the content of these thoughts is related to the task at hand (mind-wandering and task-related interferences), or (4) a mixture of these phenomena, with perhaps distinct regions of the DMN supporting distinct processes.

A number of previous fMRI and PET studies have begun to investigate the possible function of the DMN by examining correlations between brain activity and different types of conscious experiences. Some studies have inferred mind-wandering indirectly, by varying task demands to influence the probability of task-unrelated thoughts and estimating the frequency of mind-wandering episodes by collecting data outside the scanner [7], [8], [11], [14], [15], [40]. Results from these studies raise some problems of interpretation, however, because there were no online measures of conscious experience taken during scanning and it is therefore possible that the observed DMN activations are due to factors other than mind-wandering [9], [10]. Other studies have directly sampled participants' ongoing conscious experience in the scanner [16], [41]. For example, Christoff et al. [16] investigated the neural correlates of task-unrelated thoughts while the participants performed the Sustained Attention to Response Task (SART, a go/no-go task [42]) with the use of the thought-probe method [43], [44]. This method consists in interrupting the task currently being performed at various random intervals by “probes” and asking participants to report the kind of conscious experience they had in the few trials preceding the interruption. The combination of the thought-probe method with the SART has successfully been used in several studies, demonstrating a relation between off-task conscious experiences and decreased task performance (e.g., [38], [45], [46]), as well as changes in various physiological measures, such as event-related potentials, heart rate, and galvanic skin response [38], [47], [48]. In their study, Christoff et al. [16] found that task intervals for which off-task thoughts were reported were associated with increased DMN activity. Interestingly, the dorsal anterior cingulate and lateral prefrontal cortex were also engaged during off-task thoughts, suggesting a processing overlap between mind-wandering and central executive resources.

**Figure 1:**
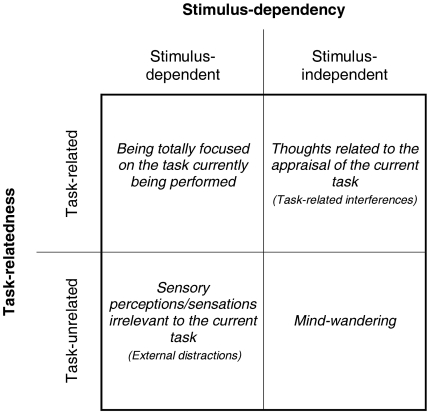
Dimensions of ongoing conscious experiences. Conceptual division of ongoing conscious experiences occurring during tasks requiring sustained externally-driven attention according to their “stimulus-dependency” and “task-relatedness” dimensions.

Although these neuroimaging findings suggest that mind-wandering is associated with the recruitment of both DMN and executive network regions, it should be noted that participants were simply asked to report whether they were totally focused on the proposed task (on-task reports) or were distracted by task-unrelated thoughts (off-task reports). This dual-choice format does not permit to clearly distinguish between mind-wandering, external distractions, and task-related interferences, such that these three types of conscious experiences might have been mixed into the same response category [9], [16]. Conceptually, the different conscious experiences that can occur while performing a task requiring focused attention and the processing of external stimuli can be characterized along two dimensions: “task-relatedness” and “stimulus-dependency.” Together, these two dimensions define four classes of conscious experiences (see Figure 1) that can be defined as (1) task-related and stimulus-dependent (i.e., being fully focused on the current task), (2) task-related and stimulus-independent (i.e., task-related interferences), (3) task-unrelated and stimulus-dependent (i.e., external distractions), (4) task-unrelated and stimulus-independent (i.e., mind-wandering).

In order to clarify the role of the DMN in mind-wandering versus unfocused external attention, the present study used a factorial design in which participants reported their conscious experiences in terms of both task-relatedness and stimulus-dependency while they performed the SART. Thus, four possibilities of responses to thought-probes were included, corresponding to the four classes of conscious experiences defined above. Our first aim was to investigate the brain regions that were specifically associated with the two defining dimensions of these conscious experiences. Furthermore, we examined the brain regions that were specifically related to (1) task-related interferences, (2) external distractions, and (3) mind-wandering, in comparison to being fully focused on the SART. A conjunction analysis was also performed to identify the brain regions that were more active during mind-wandering compared to both task-related interferences and external distractions. Finally, region of interest (ROI) analyses were performed in order to investigate whether distinct subregions of the MPFC that have been identified in previous studies (see above) are differentially sensitive to task-relatedness and stimulus-dependency. Results of these analyses demonstrate the relevance of our finer-grained conceptualization of ongoing conscious experiences for explaining variations in DMN activity. Both the “task-relatedness” and “stimulus-dependency” dimensions were indeed related to activity in several regions of the DMN, suggesting that these regions underlie cognitive processes that are active during both internal thoughts and unfocused external attention.

## Methods

### Ethics Statement

All participants gave their written informed consent to take part in the study, in line with the Declaration of Helsinki, and the study was approved by the Ethics Committee of the Medical School of the University of Liège.

### Participants

Twenty-two right-handed adults (17 women) aged between 18 and 30 years (mean age = 22 years) participated in the experiment. None of the participants had any history of neurological or psychiatric disorder.

### SART with thought-probes

The version of the SART used in the study was adapted from Christoff et al. [16]. Stimuli (numbers between 1 and 9) were presented sequentially at the center of the screen. Participants were asked to respond as fast and accurately as possible to the numbers and to withhold the response when presented with the number 3 (the target stimulus). The interstimulus interval was 1750 msec, and the duration of each stimulus (target and non-target) was 450 msec. This slow presentation rate introduced by Smallwood et al. [38] was used because it has previously been demonstrated to increase the probability of off-task reports in comparison to faster presentation rates (e.g., 1250 msec [49]). All participants signaled the presence of each non-target number via a manual finger press on a response key. Within each block of trials, target and non-target probability was randomized with the constraints that the last five stimuli of each block (i.e., stimuli presented just before the thought-probe) were non-targets. The average probability of target stimuli across blocks was 10%. Blocks were of five different durations and comprised 13, 15, 17, 19 or 21 stimuli. In total, 65 blocks were administrated in the fMRI session.

Each block was immediately followed by a thought-probe which interrupted the task. For each probe, participants were asked to characterize the conscious experience they had in the few trials prior to the probe, according to the two dimensions “task-relatedness” and “stimulus-dependency” described above (Figure 1). Four possible choices were thus provided, each being associated with a specific response key: (1) task-related and stimulus-dependent experience (i.e., on-task reports): the participant's attention and thoughts are fully focused on the task-related stimuli (i.e., the numbers); (2) task-related and stimulus-independent experience (i.e., task-related interferences reports): the participant experiences thoughts about the task that are not directly related to the numbers presented on the screen and, thus, that do not help him/her to have the best possible performance on the current ongoing trials (e.g., thoughts about task duration or about the participant's overall performance); (3) task-unrelated and stimulus-dependent experience (i.e., external distractions reports): the participant's attention is diverted by stimuli that are present in the current environment but unrelated to the task at hand (e.g., exteroceptive perceptions, such as noises, the luminance, the temperature or others features of the current environment or interoceptive sensations, such as feeling thirsty, tired or other physical sensations); (4) task-unrelated and stimulus-independent experience (i.e., mind-wandering reports): the participant has his/her attention decoupled from exteroceptive/interoceptive perceptions and is experiencing thoughts unrelated to the task at hand (e.g., thoughts about what the participant did last evening, about what he/she needs to do this evening or about what significant others could be doing now). Responses to thought-probes were self-paced. After each probe, a fixation cross was displayed on the screen for a variable duration (random normal distribution with a mean duration of 4500 ms and standard deviation of 1000 ms).

In order to familiarize participants with the thought-sampling method, they first performed a version of the SART with thought-probes outside the scanner in an interval ranging from five to one day(s) before the fMRI session. In this first session, after being presented with the instructions and before performing the SART, participants were trained in classifying ten sentences representing different conscious experiences in order to familiarize them with the four response categories, and were presented with a short example of the SART (ten numbers, two targets, and two thought-probes). Then, they performed 40 blocks of the SART. They were given a paper sheet with the instructions for responding to the probes and were told that they could keep it for the whole SART and check it in case they felt uncertain about the definition of the four response possibilities. A final training of approximately six minutes was also performed in the scanner immediately before the fMRI session, during the structural MR acquisition.

### MRI acquisition

Data were acquired on a 3 Tesla scanner (Siemens, Allegra, Erlangen, Germany) using a T2* sensitive gradient echo EPI sequence (TR = 2130 ms, TE = 40 ms, FA 90°, matrix size 64×64×32, voxel size 3.4×3.4×3.4 mm^3^). Thirty-two 3-mm thick transverse slices (FOV 22×22 cm^2^) were acquired, with a distance factor of 30%, covering the whole brain. Between 1360 and 1472 functional volumes were acquired. The first three volumes were discarded to account for T1 saturation. A structural MR scan was obtained at the beginning of the session (T1-weighted 3D MP-RAGE sequence, TR = 1960 ms, TE = 4.4 ms, FOV 23×23 cm^2^, matrix size 256×256×176, voxel size 0.9×0.9×0.9 mm). Head movement was minimized by restraining the subject's head using a vacuum cushion. Stimuli were displayed on a screen positioned at the rear of the scanner, which the subject could comfortably see through a mirror mounted on the standard head coil.

### fMRI analyses

fMRI data were preprocessed and analyzed using SPM5 (Wellcome Department of Imaging Neuroscience, http://www.fil.ion.ucl.ac.uk/spm) implemented in MATLAB (Mathworks Inc., Sherborn, MA). Functional scans were realigned using iterative rigid body transformations that minimize the residual sum of squares between the first and subsequent images. They were normalized to the MNI EPI template (voxel size: 2×2×2 mm) and spatially smoothed with a Gaussian kernel with full-width at half maximum (FWHM) of 8 mm.

For each participant, BOLD responses were modeled at each voxel, using a general linear model. Following the procedure by Christoff et al. [16], intervals of five trials (including only non-targets) preceding each probe were modeled as epoch-related responses (beginning at the onset of the fifth trials preceding the probe and ending just before the onset of the probes), according to the four kinds of responses given to the probes. The probes were also modeled as epoch-related responses (beginning at the onset of the probes and ending at their offset), using a single regressor. Target stimuli and non-target stimuli were modeled as event-related responses. The design matrix also included the realignment parameters to account for any residual movement-related effect. The canonical HRF was used. A high pass filter was implemented using a cut-off period of 256 seconds in order to remove the low-frequency drifts from the time series. Serial autocorrelations were estimated with a restricted maximum likelihood algorithm with an autoregressive model of order 1 (+white noise). Four linear contrasts were performed, looking at the effect of each kind of response given to the probes relative to baseline. The corresponding contrast images were smoothed (6-mm FWHM Gaussian kernel) in order to reduce remaining noise due to inter-subject differences in anatomical variability in the individual contrast images.

The individual summary statistics images of these four linear contrasts were then entered in second-level analyses, corresponding to random-effects models. A 2 (task-relatedness)×2 (stimulus-dependency) whole-brain voxel-wise repeated-measures analysis of variance (ANOVA) was first performed, which allowed us to examine the brain regions that are specifically related to the two dimensions of interest in this study. Then, we investigated the brain activations specific to task-related interferences relative to being fully focused on the task (task-related interferences>on-task), external distractions relative to being fully focused on the task (external distractions>on-task), and mind-wandering relative to being fully focused on the task (mind-wandering>on-task), using *t*-tests. A conjunction analysis testing the conjunction null hypothesis [50] was also performed to explore the brain regions that were more active during mind-wandering compared to both task-related interferences and external distractions [(mind-wandering>task-related interferences) ∩ (mind-wandering>external distractions)]. For a priori regions of interest, statistical inferences were corrected for multiple comparisons using Gaussian random field theory at the voxel level in a small spherical volume (radius 10 mm) around coordinates selected from the literature on the DMN and from previous studies focusing on the neural correlates of go/no-go tasks and executive control of attention. These a priori regions of interest concerned areas in MPFC (−6, 52, −2; 6, 53, −8) [8], [33], rostral MPFC (−10, 68, 20) [36], PCC/precuneus (−8, −56, 26) [33], left pIPL (−44, −74, 32; −40, −76, 40) [22], [33], left Rsp (−14, −52, 8) [33], left parahippocampal cortex (PHC; −28, −40, −12) [33], left middle and inferior temporal gyrus (MTG and ITG; −60, −24, −18; −68, −36, −4) [22], [33], right ITG (51, −13, −25) [51], bilateral middle frontal gyrus (−45, 30, 30; 48, 30, 30) [52], [53] and left anterior IPL (−52, −49, 47) [35]. The coordinates reported in the studies by Mason et al., Toro et al., and Weisman et al. [8], [51], [52] were transformed to the MNI space using a nonlinear transformation (http://imaging.mrc-cbu.cam.ac.uk/imaging/MniTalairach). For completeness, the supplementary tables also list other regions that survived a threshold of *p*<0.001, uncorrected for multiple comparisons with a minimum cluster size of 15 voxels, but these regions are not discussed further.

Finally, ROI analyses were performed in order to investigate the possible distinction between a rostral portion versus a more caudal portion of the MPFC. Gilbert et al. [36] found that a rostral region of the MPFC (−10, 68, 20) was associated with attention towards external stimuli, whereas an adjacent but more caudal—and somewhat more dorsal—region of MPFC (−8, 54, 30), highly similar to the one associated with mind-wandering [8], [10], was related to mentalizing. In this study, an ROI analysis using coordinates of these two regions was performed in order to investigate whether they are differentially sensitive to the two dimensions of conscious experiences investigated here. For each participant, parameter estimates (mean beta weights) were derived from the rostral MPFC (averaged over all voxels within a 4 mm radius sphere of the peak voxel: −10, 68, 20) and the more caudal MPFC (averaged over all voxels within a 4 mm radius sphere of the peak voxel: −8, 54, 30) for each of the four conditions (relative to the baseline). A 2 (task-relatedness)×2 (stimulus-dependency)×2 (regions) repeated measures ANOVA was then performed on the parameter estimates.

## Results

### Behavioral data

Mean response time for non-targets was 405 msec (*SD* = 112). Response withholding accuracy for targets was 62.28% (*SD* = 16.94). Regarding thought-probes, participants reported being on-task for 31.59% (*SD* = 14.27) of probes, task-related interferences for 22.13% (*SD* = 7.62) of probes, external distractions for 25.44% (*SD* = 10.04) of probes, and mind-wandering for 20.83% (*SD* = 11.17) of probes.

Next, we examined the impact of the four types of reports on response times (RTs) for the five non-target stimuli preceding each probe. A 2 (task-relatedness)×2 (stimulus-dependency) repeated measures ANOVA on mean RTs was performed. Mean values and standard deviations are detailed in Table 1. Analysis on mean RTs did not demonstrate any main effect of task-relatedness [*F*(1,21) = 0.66; *p* = 0.42; partial *η^2^* = 0.03] or of stimulus-dependency [*F*(1,21) = 0.42; *p* = 0.52; partial *η^2^* = 0.02], and the interaction was not significant either [*F*(1,21) = 0.25; *p* = 0.62 ; partial *η^2^* = 0.01]. A second ANOVA was conducted on the variability of RTs (as assessed by the coefficient of variation; the ratio of the standard deviation σ to the mean μ): we observed a main effect of task-relatedness [*F*(1,21) = 4.39; *p*<0.05; partial *η^2^* = 0.17], no main effect of stimulus-dependency [*F*(1,21) = 1.90; *p* = 0.18; partial *η^2^* = 0.08], and a significant interaction [*F*(1,21) = 8.71; *p*<0.01; partial *η^2^* = 0.29], indicating that reports of being completely focused on the task were preceded by more stable RTs than the three other classes of reports (all *p*s<0.05; Tukey's HSD test). There was no significant difference between the three other kinds of reports.

**Table 1:** Mean performance for each block according to the responses given to the though-probes.

	Mean RTs for the 5 last non-targets	Mean CVs for the 5 last non-targets	Mean percentage of errors to the targets
On-task	401 (108)	14.89 (5.00)	27.62 (17.59)
TRIs	408 (128)	18.55 (6.70)	46.18 (20.51)
EDs	410 (127)	19.29 (7.40)	46.47 (16.06)
Mind-wandering	411 (136)	18.30 (8.67)	38.12 (20.98)

Note: Standard deviations from the mean are presented in brackets. RTs: response times: CVs: coefficients of variation; TRIs: Task-related interferences; EDs: External distractions. Mean RTs are presented in msec.

We also examined the effect of the four types of reports on response accuracy for the whole block of trials. We performed a 2 (task-relatedness)×2 (stimulus-dependency) repeated measures ANOVA on the number of errors to the target stimuli. Mean values and standard deviations are detailed in Table 1. The ANOVA demonstrated a main effect of task-relatedness [*F*(1,21) = 7.74; *p*<0.05; partial *η^2^* = 0.27], no main effect of stimulus-dependency [*F*(1,21) = 3.08; *p* = 0.09; partial *η^2^* = 0.13], and a significant interaction [*F*(1,21) = 28.10; *p*<0.01; partial *η^2^* = 0.57], indicating that reports of being completely focused on the task were preceded by fewer errors to the targets than the three other kinds of reports (all *p*s<0.05; Tukey's HSD test). There was no significant difference between the three other types of reports.

In order to examine whether the time spent on the SART had an impact on the distribution of responses to thought-probes, an index of the effect of time on probe responses was calculated for each of the four kinds of reports according to the following formula: number of reports for the 32 last blocks of the SART – number of reports for the 32 first blocks of the SART. Mean values of this index were −1.86 (*SD* = 4.03) for reports of being fully focused on task, −0.09 (*SD* = 3.07) for task-related interferences reports, 1.73 (*SD* = 4.46) for external distractions reports, and 0.23 (*SD* = 3.05) for mind-wandering reports. Examination of the 95% confidence intervals revealed that the frequency of reports of being fully focused on task decreased with time on the task (−3.65; −0.08). None of the other kinds of reports significantly increased/decreased in frequency with time on the SART: task-related interferences (−1.45; 1.27), external distractions (−0.25; 3.71), and mind-wandering (−1.13; 1.58). Combining these indexes revealed that the time spent on the SART had no effect on task-unrelated reports (*M* = 1.95, *SD* = 4.72, *CI* = −0.14; 4.05) and stimulus-independent reports (*M* = 0.14, *SD* = 4.39, *CI* = −1.81; 2.08). These results indicate that the time spent on the SART had minor impact on the distribution of responses to the thought-probes in this study and thus cannot fully account for the fMRI results.

Overall, the behavioral data on SART performance are consistent with previous studies using a similar method [46], [54] and provide a behavioral validation of the finer-grained thought-probe method used in this study to assess the four classes of conscious experiences of interest. With regards to the effect of time on task, it should be noted that contrary to the present study, several previous studies using thought-probes found that the frequency of off-task reports increased with the time spent on a task [38], [46], [54], [55]. The absence of time effect in this study could be explained by the fact that participants were extensively trained with the SART both in the days preceding and immediately before the fMRI session, which could have reduced the effect of time on task during the functional acquisition.

### fMRI data

#### Brain regions associated with task-relatedness and stimulus-dependency

**Figure 2:**
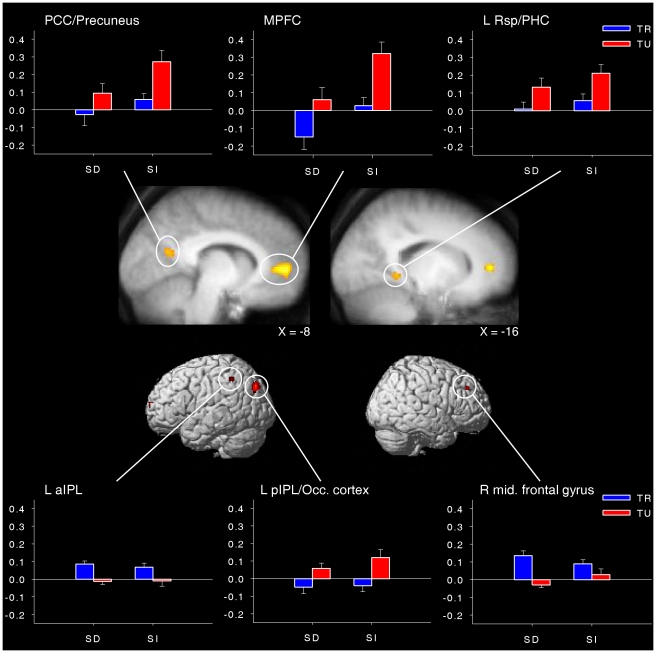
Brain areas associated with the main effect of task-relatedness. Regions are displayed at *p*<0.001, uncorrected for multiple comparisons with a minimum cluster size of 15 voxels. Bar graphs illustrate the mean parameter estimates (± standard error of the mean) for each cluster. SD = stimulus-dependent, SI = stimulus-independent, TR = task-related, TU = task-unrelated, L = left, R = right, MPFC = medial prefrontal cortex, PCC = posterior cingulate cortex, aIPL = anterior inferior parietal lobe, pIPL = posterior inferior parietal lobe, Occ. cortex = occipital cortex, Rsp = restrosplenial cortex, PHC = parahippocampal cortex.

First, in order to examine the brain regions associated with the two dimensions of conscious experiences of interest, a 2 (task-relatedness)×2 (stimulus-dependency) whole-brain voxel-wise ANOVA was performed. The main effect of task-relatedness revealed clusters of activation in the medial prefrontal cortex (MPFC), the posterior cingulate cortex (PCC)/precuneus, the left posterior inferior parietal lobule (pIPL)/occipital cortex, the left anterior inferior parietal lobule (aIPL), the right middle frontal gyrus and the left parahippocampal cortex (PHC)/restrosplenial cortex (Rsp). Examination of the parameter estimates for each condition revealed that all of these brain regions were more active during intervals preceding task-unrelated reports (i.e., mind-wandering and external distractions) than during intervals preceding task-related reports (i.e., on-task and task-related interferences), except for the left aIPL and the right middle frontal gyrus for which the opposite effect was observed (Figure 2 and Table 2; see also Table S1 for clusters of activation located outside a priori areas of interest). Thus, in accordance with our hypotheses, DMN regions were associated with task-unrelated reports, whereas regions outside the DMN that have been previously linked with executive control and successful performance during go/no-go tasks (the left aIPL and right middle frontal gyrus) were associated with task-related reports. Importantly, not every region of the DMN was associated with task-unrelated reports, as no evidence of activation in lateral temporal regions was found in this particular analysis.

**Table 2:** Brain regions associated with the main effects of task-relatedness and stimulus-dependency in the whole-brain ANOVA.

	MNI coordinates				
	*x*	*y*	*z*	Voxels	*F*
***Main effect of task-related.***					
MPFC	0	58	−2	634	24.11
PCC/Precuneus	−8	−62	20	50	13.41
L pIPL/occipital cortex	−38	−80	36	100	15.20
L Rsp, PHC	−16	−48	0	26	12.43
R middle frontal gyrus	48	38	36	20	14.94
L aIPL	−60	−48	48	18	14.08
***Main effect of stimulus-dep.***					
MPFC	2	58	−2	1206	18.37
Rostral MPFC	−2	70	20	103	16.17
PCC/Precuneus	−2	−50	22	972	19.37
L pIPL	−44	−72	48	287	16.35
L inf./mid. temporal gyrus	−64	−22	−26	480	22.54
R inf./mid. temporal gyrus	52	−14	−32	334	21.89
L middle temporal gyrus	−64	−44	−8	308	16.94
L inferior frontal gyrus	−52	26	24	191	12.35

Note: All regions are significant at *p*<0.05, corrected for multiple comparisons at the voxel level over small volumes of interest (see Methods for details). L = left, R = right, MPFC = medial prefrontal cortex, PCC = posterior cingulate cortex, pIPL = posterior inferior parietal lobe, aIPL = anterior inferior parietal lobe, Rsp = restrosplenial cortex, PHC = parahippocampal cortex.

**Figure 3:**
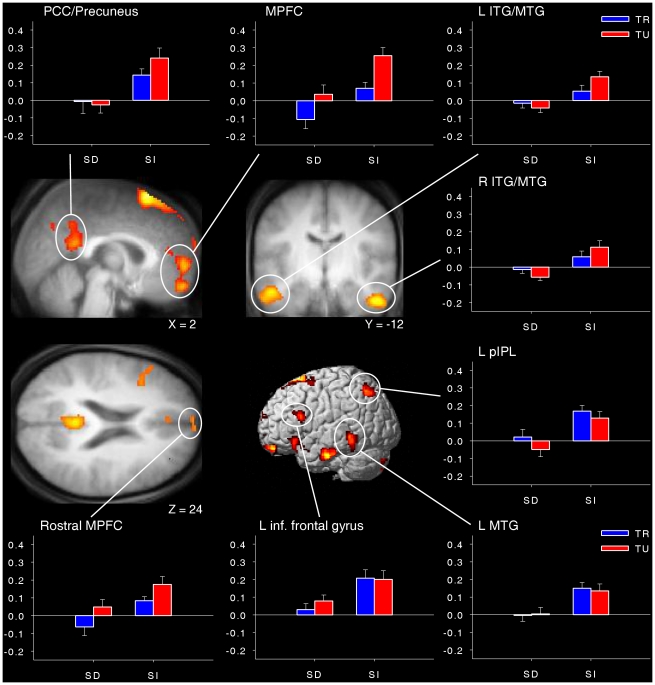
Brain areas associated with the main effect of stimulus-dependency. Regions are displayed at *p*<0.001, uncorrected for multiple comparisons with a minimum cluster size of 15 voxels. Bar graphs illustrate the mean parameter estimates (± standard error of the mean) for each cluster. SD = stimulus-dependent, SI = stimulus-independent, TR = task-related, TU = task-unrelated, L = left, R = right, MPFC = medial prefrontal cortex, PCC = posterior cingulate cortex, pIPL = posterior inferior parietal lobe, ITG = inferior temporal gyrus, MTG = middle temporal gyrus.

The main effect of stimulus-dependency was also associated with clusters of activations in the MPFC, extending more ventrally and anteriorly than for the main effect of task-relatedness, as well as in the PCC/precuneus and the left pIPL (with the latter region being located somewhat more anteriorly than the region observed for the main effect of task-relatedness). Other clusters of activations included the bilateral inferior/middle temporal gyrus (ITG/MTG), the left MTG and the left inferior frontal gyrus. Examination of the parameter estimates revealed that all of these regions were more active in intervals preceding stimulus-independent reports (i.e., mind-wandering and task-related interferences) than in intervals preceding stimulus-dependent reports (i.e., on-task and external distractions; Figure 3 and Table 2; see also Table S1 for clusters of activation located outside a priori areas of interest). These results indicate that the core DMN regions associated with task-unrelated reports are also associated with stimulus-independent reports. They also extend the findings of previous studies that used simpler dual-choice thought-probes and permit to rule out a specific implication of the DMN in either one or the other of the two dimensions of interest here. In addition, the main effect of stimulus-dependency revealed that the lateral temporal regions (also part of the DMN) and the left inferior frontal gyrus were specifically associated with stimulus-independent reports.

Finally, although not of primary interest here, the whole-brain ANOVA also showed a cross-over interaction effect in some brain regions such as the right middle frontal gyrus, the right ITG, and the left anterior PHC (Table S1).

#### Brain regions associated with task-related interferences, external distractions, and mind-wandering

**Figure 4:**
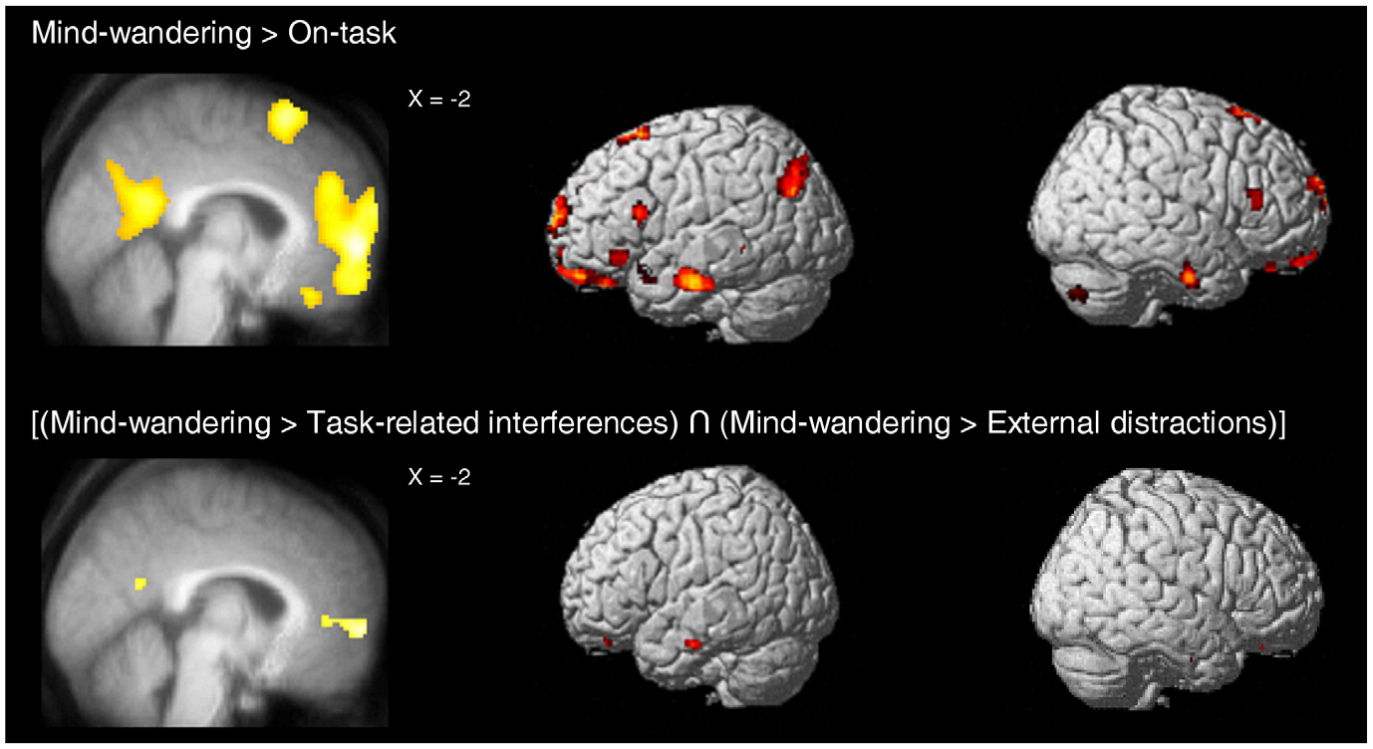
Brain areas associated with mind-wandering. The brain areas associated with mind-wandering compared to being fully focused on task are shown in the superior panels. The brains areas associated with mind-wandering compared to both task-related interferences and external distractions (conjunction analysis) are shown in the inferior panels. Regions are displayed at *p*<0.001, uncorrected for multiple comparisons with a minimum cluster size of 15 voxels.

The introduction of thought-probes providing four possibilities of response permitted us to examine the brain regions specifically associated with task-related interferences, external distractions, and mind-wandering in comparison to being fully focused on the SART. The direct contrast between task-related interferences and being on-task revealed a cluster of activation in the right ventral MPFC (Table 3). The direct contrast between external distractions and being on-task revealed a small activation in the left dorsal MPFC (Table 3). Finally, the direct contrast of mind-wandering with being on-task revealed clusters of activation in the MPFC, the PCC extending into the left Rsp and PHC, the left pIPL, and the bilateral ITG/MTG (Figure 4, Table 3; see also Table S2 for clusters of activation located outside a priori areas of interest).

**Table 3:** Brain regions associated with task-related interferences, external distractions, and mind-wandering compared to being fully focused on task.

	MNI coordinates				
	*x*	*y*	*z*	Voxels	*t*
***TRIs>on-task***					
R ventral MPFC	14	54	−12	17	3.32
***EDs>on-task***					
L dorsal MPFC	−12	60	18	7	3.47
***Mind-wandering>on-task***					
MPFC	0	58	0	4649	5.83
L PCC/Precuneus, Rsp, PHC	−2	−56	22	1766	4.57
	−14	−46	4		3.92
	−20	−40	−6		3.55
L pIPL	−38	−72	34	475	3.97
L inf./mid. temporal gyrus	−56	−16	−22	523	4.21
R inf./mid temporal gyrus	50	−12	−32	181	3.74

Note: All regions are significant at *p*<0.05, corrected for multiple comparisons at the voxel level over small volumes of interest (see Methods for details). TRIs = task-related interferences, EDs = external distractions, L = left, R = right, MPFC = medial prefrontal cortex, PCC = posterior cingulate cortex, pIPL = posterior inferior parietal lobe, Rsp = restrosplenial cortex, PHC = parahippocampal cortex.

Considering that task-related interferences and external distractions were associated with activations in distinct subregions of MPFC (i.e., ventral versus dorsal MPFC) compared to being on-task (see Table 3), we also performed direct contrasts between these two kinds of reports to further examine these possible differences regarding MPFC activations. No brain region was more active during external distractions compared to task-related interferences. The reverse contrast (task-related interferences>external distractions) revealed clusters of activation in the left MTG (MNI coordinates of peak voxel: −68, −44, −10; *t* = 3.60, *p_SVC_*<0.05) and in the two regions previously associated with task-related conscious experiences in the whole brain ANOVA: the left aIPL (MNI coordinates of peak voxel: −48, −56, 52; *t* = 4.05, *p_SVC_*<0.05) and the right middle frontal gyrus (MNI coordinates of peak voxel: 48, 36, 38; t = 3.69, *p_SVC_*<0.05); see also Table S3 for clusters of activation located outside a priori areas of interest. We did not find any subregion within MPFC that would be more strongly associated with one or the other category of reports.

#### Brain regions showing higher activity during mind-wandering compared to both task-related interferences and external distractions

The common involvement of DMN regions in both task-unrelated reports and stimulus-independent report, as well as the absence of interaction effect for these regions and the results of the direct contrasts suggest an additive effect of the “task-relatedness” and “stimulus-dependency” dimensions of conscious experiences on DMN activity. Mind-wandering would be associated with the highest level of DMN activity, task-related interferences and external distractions with intermediate levels of DMN activity, and being fully focused on the SART with the lowest level of activity in this network. We then conducted a conjunction analysis testing the conjunction null hypothesis: [(mind-wandering>task-related interferences) ∩ (mind-wandering>external distractions)] to confirm that DMN regions are indeed more active during mind-wandering compared to both task-related interferences and external distractions. This conjunction analysis revealed clusters of activations in the MPFC, the PCC/precuneus, and left MTG (Figure 4, Table 4; see also Table S4 for clusters of activation located outside a priori areas of interest). These results thus confirm that the common implication of midline DMN regions in both task-unrelated and stimulus-independent reports results from an additive effect of these two dimensions of conscious experiences on DMN activity.

**Table 4:** Brain regions more active during mind-wandering compared to both task-related interferences and external distractions (conjunction analysis).

	MNI coordinates				
	*x*	*y*	*z*	Voxels	*t*
MPFC	0	60	−2	310	3.79
PCC/Precuneus	−4	−60	22	25	3.35
L middle temporal gyrus	−56	−16	−22	27	3.28

Note: All regions are significant at *p*<0.05, corrected for multiple comparisons at the voxel level over small volumes of interest (see Methods for details). L = left, MPFC = medial prefrontal cortex, PCC = posterior cingulate cortex.

#### ROIs analyses

**Figure 5:**
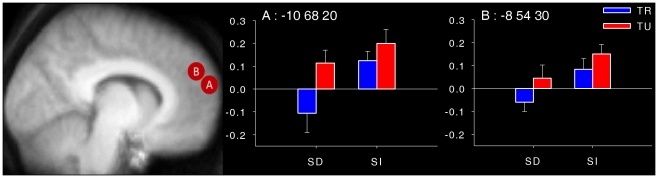
Mean parameters estimates for a rostral portion versus a more caudal portion of the MPFC. Mean parameter estimates (averaged over all voxels within a 4-mm radius of the peak voxel) for each of the four conditions (relative to the baseline) in two regions of the medial prefrontal cortex. Error bars represent the standard error of the mean. SD = stimulus-dependent, SI = stimulus-independent, TR = task-related, TU = task-unrelated.

Finally, because (1) our clusters of activation in the MPFC were rather extended and (2) it has been suggested that closely juxtaposed portions of the MPFC may perform different cognitive functions, a 2 (task-relatedness)×2 (stimulus-dependency)×2 (regions) repeated measures ANOVA was performed on the parameter estimates for two subregions of the MPFC that have been differentially linked to external attention and mind-wandering/mentalizing in previous studies [8], [9], [10], [36]. As illustrated in Figure 5, the ANOVA demonstrated a main effect of task-relatedness [*F*(1,21) = 5.39; *p*<0.05; partial *η^2^* = 0.20], revealing that the two regions showed higher activity for task-unrelated reports, and a main effect of stimulus-dependency [*F*(1,21) = 11.18; *p*<0.01; partial *η^2^* = 0.35], revealing that the two regions showed higher activity for stimulus-independent reports. The interaction between task-relatedness and stimulus-dependency was not significant [*F*(1,21) = 1.61; *p* = 0.22; partial *η^2^* = 0.07], indicating an additive effect of these two dimensions. Furthermore, the main effect of regions was not significant [*F*(1,21) = 1.57; *p* = 0.22; partial *η^2^* = 0.07], and none of the interaction effects involving regions were significant: task-relatedness×regions [*F*(1,21) = 0.65; *p* = 0.43; partial *η^2^* = 0.03], stimulus-dependency×regions [*F*(1,21) = 0.16; *p* = 0.69; partial *η^2^*<0.01], and task-relatedness×stimulus-dependency×regions [*F*(1,21) = 0.47; *p* = 0.50; partial *η^2^* = 0.02], indicating that the two ROIs showed similar patterns of activations as a function of the two dimensions of conscious experience under investigation here. Overall, these results are thus in line with the results of the whole brain analyses, showing an additive effect of “task-relatedness” and “stimulus-dependency” on MPFC activity.

## Discussion

Previous neuroimaging studies suggest that mind-wandering is associated with increased DMN activity. However, in these studies, participants were simply asked to report whether they were totally focused on the proposed task (on-task reports) or were distracted by task-unrelated thoughts (off-task reports), which does not permit to clearly distinguish between mind-wandering, external distractions, and task-related interferences. The present study aimed at clarifying this issue by investigating the neural correlates of the various kinds of conscious experiences that can occur while participants perform a task requiring sustained focused attention and the cognitive processing of external stimuli. Four classes of conscious experiences (being fully focused on the task, task-related interferences, external distractions, and mind-wandering) that varied along two dimensions (“task-relatedness” and “stimulus-dependency”) were sampled using newly designed thought-probes while participants performed the Sustained Attention to Response Task (SART [42]).

We found that reports of task-unrelated conscious experiences were associated with increased activity in the MPFC, the PCC/precuneus, and the pIPL, all regions of the DMN [1], [3], [5]. Importantly, increased activity in midline DMN regions was also associated with reports of stimulus-independent conscious experiences, independently of whether the content of these thoughts was related to the SART or not. Furthermore, we found that the two dimensions (i.e., task-relatedness and stimulus-dependency) had additive effects on the activity of midline DMN regions. External distractions (i.e. stimulus-dependent and task-unrelated reports) and task-related interferences (i.e., stimulus-independent and task-related reports) were associated with intermediate levels of activity, whereas mind-wandering (i.e., stimulus-independent and task-unrelated reports) and on-task reports (i.e., stimulus-dependent and task-related reports) were respectively associated with the highest and lowest degree of activity in the MPFC and the PCC/precuneus. Notably, an analysis of variance was performed on the parameter estimates of the BOLD response extracted from a rostral portion of the MPFC previously linked with attention toward external stimuli [36] and a more caudal portion of the MPFC previously linked with mentalizing and mind-wandering [8], [36]. This analysis revealed significant main effects of task-relatedness and stimulus-dependency but no significant main effect of regions and no significant interaction effect, therefore demonstrating similar additive effects of the two dimensions of conscious experiences for both regions of MPFC. Finally, we found (1) that activity in the lateral temporal lobe—also part of the DMN [1], [3], [5]—was related to stimulus-independent reports but not to task-unrelated reports, (2) that different parts of the lateral prefrontal cortex were linked to task-related reports and stimulus-independent reports, and (3) that the aIPL was specifically activated in relation to task-related reports.

These results are important because they help reconcile different proposals that have been made regarding the functional role of the DMN. For instance, Gusnard and Raichle [32] have proposed that DMN activity reflects a state of unfocused attention in which both internal thoughts and stimulus from the external world are gathered and monitored (i.e., task-unrelated conscious experiences), whereas others [12], [13] have linked DMN activity to a general shift of attention from stimuli in the external environment toward internal thoughts (i.e., stimulus-independent conscious experiences). The present findings suggest that these two views are not mutually exclusive and show that both the “task-relatedness” and “stimulus-dependency” dimensions of conscious experiences are related to DMN activity. Indeed, these two dimensions had additive effects on DMN activity, such that mind-wandering episodes were associated with the highest degree of DMN activity but attention towards external task-unrelated stimuli (i.e., external distractions) and thoughts related to the appraisal of the SART (i.e., task-related interferences) were also associated with higher DMN activity compared to being fully focused on the task. These results thus highlight the importance of simultaneously considering the task-related and stimulus-dependent dimensions of ongoing conscious experience when attempting to link DMN activity to particular cognitive functions.

The MPFC, PCC/precuneus, and pIPL were engaged during both task-unrelated and stimulus-independent conscious experiences. This result suggests that these regions underlie cognitive processes active during both unfocused attention toward external stimuli and internal thoughts. A possibility is that increased activity in these DMN regions (especially midline regions) reflects the engagement of cognitive processes involved in monitoring the self-relevance of the ongoing content of consciousness [56], [57], [58], [59], independently of whether it refers to current sensory input or internal thought. Previous research has shown that self-relevant thoughts are an important part of the spontaneous cognitive activity at rest and that reports of these thoughts correlate with activity in midline DMN regions [60]. The MPFC and PCC/precuneus have also been implicated in self-referential judgments of external stimuli [56], [61], [62], and activity in these regions is parametrically modulated by the degree of self-relatedness of the stimuli [63]. Furthermore, a recent study has shown that activity in the hubs of the DMN, namely the MPFC and PCC, correlates with the use of self-referential strategies when making self-referential or nonpersonal semantic judgments [33]. Given that the content of many mind-wandering episodes is personally significant [11], [64] and often involves thoughts about one's most important current concerns [65], [66], the larger activation of the MPFC and PCC/precuneus that we observed during mind-wandering could be related to the higher degree of self-relevance of these thoughts in comparison to task-related interferences and external distractions. Further research should be conducted to assess this possibility.

The moderate level of DMN activity related to external distractions and task-related interferences might suggest that both are intermediate states preceding the occurrence of mind-wandering. There is some behavioral evidence that the occurrence of mind-wandering is not an all or nothing phenomenon but follows a gradual temporal sequence [49], [67]. Regarding brain activity, some authors have suggested that increased DMN activity does not necessarily reflect mind-wandering until a certain threshold of activation is reached [68]. For these authors, the DMN could be involved in a continuous generation of predictions of the future based on the content of long-term memory [1], [69], [70]. The function of these predictions would be to guide our actions according to the particular context in which we behave [71], [72]. It might be that mind-wandering is the by-product of the continuous generation of predictions underlain by the DMN [68]. Thus, the intermediate level of DMN activity in association with external distractions and task-related interferences could prefigure the occurrence of mind-wandering, indicating that the cognitive processes involved in the generation of predictions are more active than when one is fully focused on the SART but not yet sufficiently active to uncouple attention from both external stimuli and the current task. However, whether mind-wandering episodes are always preceded by either task-related interferences or external distractions has not yet been precisely investigated. Studies focusing more specifically on the temporal sequence of brain activity and conscious experiences leading to mind-wandering should be conducted to assess this question.

Another interpretation of our findings would be that the increased midline DMN activity, and especially MPFC activity [9], [73], related to mind-wandering in comparison to task-related interferences and external distractions reflects an increased effort to reorient attention toward the SART. However, the fact that these regions are usually more active when there is no particular task to perform (e.g., at rest) than during cognitive tasks argues against this possibility. Furthermore, Christoff et al. [16] recently found that midline DMN regions are more active when participants are unaware rather than aware that their mind is currently off-task. If activity in these regions reflected efforts to refocus on the task being performed, the opposite pattern of activity should have been observed, as awareness would presumably favor the recruitment of cognitive processes to reorient attention on task.

Among the DMN regions, the lateral temporal lobes were linked to stimulus-independent reports but not to task-unrelated reports. This finding indicates that distinct subregions within the DMN are related to specific dimensions of conscious experiences, which is in agreement with recent studies demonstrating the heterogeneity of the DMN and its fractionation in subcomponents that might support different cognitive processes [33], [34], [35]. The lateral temporal lobes have been implicated in various kinds of tasks involving a decoupling of attention from the immediate environment, such as mental time travel in the past and the future or the production of conceptual judgments about presented words [21], [22], [74]. More specifically, these regions may be involved in the processing of semantic and conceptual information that is used in judgment making and memory construction [74], [75], [76]. The activation of lateral temporal regions in relation to stimulus-independent conscious experiences in this study might therefore reflect the access to the personal and general semantic knowledge that constitutes, in part, the content of these thoughts.

Stimulus-independent reports were also characterized by activity in the inferior frontal gyrus, a region that has been linked to executive functions and goal-directed activities [77], [78]. Two different explanations could account for this result. First, activation in this region might be related to attempts to control the content of consciousness, and thus to suppress thoughts that impair current task performance [79]. The lateral prefrontal cortex has indeed been linked to thought suppression processes [80]. However, it typically activates in a sustained manner during periods requiring cognitive control and no evidence of its transient activation has been found in situations where participants actively need to suppress a particular thought from working memory. As cognitive control was required throughout the whole SART, a specific increase of activity in the lateral prefrontal cortex in only some trials is difficult to explain. The second explanation is that stimulus-independent thoughts are a demanding mental activity that entails the recruitments of brain regions involved in executive functions [16], [43]. This suggestion is supported by behavioral studies which demonstrated that the frequency of stimulus-independent thoughts decreases with increasing executive demands of the task currently being performed, suggesting that executive resources can be recruited either by the current task or by stimulus-independent thoughts [81], [82]. As previously mentioned, Christoff et al. [83] have found that off-task thoughts not only recruit DMN regions but also executive network regions, suggesting a processing overlap between mind-wandering and central executive resources. Furthermore, a recent fMRI study has shown that the lateral prefrontal cortex can couple its activity with the DMN during internalized autobiographical planning [84]. Interestingly, a possible function of stimulus-independent thoughts may precisely be to prepare for upcoming events and to manage one's current concerns [43], [54]. The activation of the inferior frontal gyrus during stimulus-independent conscious experiences might therefore be related to executive processes involved in the management of personal goals and concerns, rather than processes involved in the suppression of one's thoughts.

Finally, in contrast to the posterior lateral prefrontal activation related to stimulus-independent reports, a more anterior region of the middle frontal gyrus and the aIPL were specifically activated in relation to task-related reports. The middle frontal gyrus is usually considered as being part of the dorsal attention network, a set of dorsal brain regions supporting top-down focused attention toward external stimuli [18], [51], [85]. Regarding the aIPL, several studies have recently demonstrated that this region commonly co-activates with those involved in executive functions and goal-directed activities, such as the middle frontal gyrus and the anterior cingulate cortex [23], [35], [86], [87], [88]. Furthermore, meta-analyses performed on neuroimaging studies involving go/no-go tasks have shown that successful performance on these tasks is associated with activations in a frontoparietal network comprising the middle frontal gyrus and aIPL [89], [90], [91]. Our results are in agreement with these previous findings, as increased activity in these two regions were related to the highest rate of attention directed toward the SART, both subjectively with thought-probe reports and objectively with task performance (i.e., less errors and less variability of RTs for task-related reports). These results also further demonstrate the validity of subjective reports in neuroimaging studies and suggest that this kind of procedure is not only reliable to study mind-wandering and other forms of task-unrelated attention but could also be useful to study on-task thoughts, such as the various strategies that can be used in problem solving, multitasking or encoding complex material, for instance (see [92] for a more extended discussion on this topic).

To conclude, this study demonstrates that task-relatedness and stimulus-dependency are both related to DMN activity. Notably, an additive effect of these two dimensions of conscious experiences was demonstrated for the MPFC and PCC/precuneus. On the other hand, other DMN regions were specifically related to only one dimension, with activity in lateral temporal regions being solely related to stimulus-independent reports. These results have two broad implications. First, they reveal the necessity to take both dimensions into account when attempting to determine the functional role of the DMN. This could help resolve controversies as to the role of the DMN in internal versus external attention [8], [9], [10]. Second, the present results further demonstrate that the DMN is not unitary and suggest that its constituent regions might fulfill different cognitive functions [33], [34], [35]. We tentatively propose that the gradual recruitment of the MPFC and PCC/precuneus with task-unrelatedness and stimulus-independence reflects increasing self-referential processes [56], [57], [58], [59], [60] or processes involved in the generation of predictions of the future that are based on long-term memory content [1], [19], [69], [72]. These two possibilities are not mutually exclusive, and it has recently been demonstrated that MPFC engagement during memory retrieval is stronger when the retrieved material is self-referential [93]. On the other hand, the activity in lateral temporal regions in relation to stimulus-independent conscious experiences could reflect the access to the semantic information and knowledge that are used to construct these thoughts [74], [75]. Further investigations should be conducted to assess these possibilities in more detail. The study of ongoing conscious experience has been relatively overlooked in neuroscience, despite having a long history of research in cognitive psychology [43], [94], [95]. The present findings (see also [16], [41]) provide evidence that it is nonetheless feasible and may prove fruitful in elucidating the precise functional role of the DMN in human cognition.

## Supporting Information

Table S1 Brain regions (outside a priori areas of interest) associated with the main effects of task-relatedness and stimulus-dependency, as well as the cross-over interaction effect in the whole-brain ANOVA.
(DOC)

Table S2 Brain regions (outside a priori areas of interest) associated with task-related interferences and mind-wandering compared to being fully focused on task.
(DOC)

Table S3 Brain regions (outside a priori areas of interest) more active during task-related interferences compared to external distractions.
(DOC)

Table S4 Brain regions (outside a priori areas of interest) more active during mind-wandering compared to both task-related interferences and external distractions (conjunction analysis).
(DOC)
