# Supplementary material for: Neural Correlates of Ongoing Conscious Experience: Both Task-Unrelatedness and Stimulus-Independence Are Related to Default Network Activity
Source: PLoS One. 2011 Feb 14;6(2):e16997. doi: 10.1371/journal.pone.0016997 (PMC3038939; doi:10.1371/journal.pone.0016997)
Supplement: Table S1 — Brain regions (outside a priori areas of interest) associated with the main effects of task-relatedness and stimulus-dependency, as well as the cross-over interaction effect in the whole-brain ANOVA. (DOC) [file pone.0016997.s001.doc]

Table S1. Brain regions (outside a priori areas of interest) associated with the main effect of task-relatedness and stimulus-dependency, as well as the cross-over interaction effect in the whole-brain ANOVA

|  | MNI coordinates | | |  |  |
| --- | --- | --- | --- | --- | --- |
|  | *x* | *y* | *z* | Voxels | *F* |
| ***Main effect of task-related.*** |  |  |  |  |  |
| L inf. aIC/extended amygdala | -28 | 12 | -16 | 84 | 18.24 |
| ***Main effect of stimulus-dep.*** |  |  |  |  |  |
| R middle temporal gyrus | 64 | -40 | -6 | 50 | 16.76 |
| R inferior frontal gyrus | 62 | 28 | 18 | 50 | 16.33 |
| Pre-SMA/L sup. frontal gyrus | -2 | 24 | 68 | 1258 | 30.23 |
|  | -26 | 16 | 68 |  | 19.68 |
|  | -36 | 6 | 66 |  | 19.07 |
| R orbital frontal gyrus | 26 | 22 | -20 | 20 | 14.14 |
| L inferior/orbital frontal gyrus | -44 | 26 | -24 | 222 | 18.04 |
|  | -54 | 24 | -14 |  | 17.15 |
| R middle frontal gyrus | 42 | 18 | 56 | 59 | 16.56 |
| Cuneus | 4 | -104 | -2 | 18 | 13.57 |
| L inf. aIC/extended amygdala | -28 | 8 | -24 | 174 | 19.08 |
| L thalamus | -12 | -28 | -6 | 262 | 16.77 |
| L cerebellum | -38 | -86 | -36 | 32 | 16.41 |
| R cerebellum | 46 | -78 | -42 | 217 | 22.21 |
| ***Cross-over interaction effect*** |  |  |  |  |  |
| R middle frontal gyrus | 36 | 24 | 52 | 153 | 18.20 |
| R inferior temporal gyrus | 50 | 2 | -40 | 78 | 15.18 |
| L anterior PHC | -26 | -10 | -34 | 20 | 13.27 |

Note: All regions are significant at *p* < 0.001, uncorrected for multiple comparisons with a minimum cluster size of 15 voxels. L = left, R = right, PHC = parahippocampal cortex, aIC = anterior insular cortex, Pre-SMA = pre-supplementary motor area.
