# Supplementary material for: Neural Correlates of Ongoing Conscious Experience: Both Task-Unrelatedness and Stimulus-Independence Are Related to Default Network Activity
Source: PLoS One. 2011 Feb 14;6(2):e16997. doi: 10.1371/journal.pone.0016997 (PMC3038939; doi:10.1371/journal.pone.0016997)
Supplement: Table S2 — Brain regions (outside a priori areas of interest) associated with task-related interferences and mind-wandering compared to being fully focused on task. (DOC) [file pone.0016997.s002.doc]

Table S2. Brain regions (outside a priori areas of interest) associated with task-related interferences and mind-wandering compared to being fully focused on task

|  | MNI coordinates | | |  |  |
| --- | --- | --- | --- | --- | --- |
|  | *x* | *y* | *z* | Voxels | *t* |
| ***TRIs > on-task*** |  |  |  |  |  |
| L ventral MPFC | -10 | 54 | -18 | 15 | 3.58 |
| Pre-SMA/L sup. frontal gyrus | -2 | 22 | 68 | 376 | 4.27 |
|  | -20 | 14 | 66 |  | 3.35 |
| L superior frontal gyrus | -30 | 4 | 70 | 82 | 3.92 |
| R cerebellum | 16 | -98 | -34 | 110 | 3.76 |
| ***Mind-wandering > on-task*** |  |  |  |  |  |
| L inferior frontal gyrus | -40 | 16 | 20 | 162 | 3.94 |
| R inferior frontal gyrus | 62 | 30 | 12 | 86 | 3.88 |
| L middle temporal gyrus | -48 | -44 | -2 | 21 | 3.30 |
| L anterior PHC | -26 | -8 | -40 | 55 | 3.67 |
| Precuneus | -6 | -50 | 40 | 42 | 3.36 |
| Pre-SMA | -2 | 22 | 64 | 515 | 4.92 |
| L superior frontal gyrus | -26 | 14 | 66 | 77 | 3.71 |
| L inferior/orbital frontal gyrus | -42 | 30 | -10 | 94 | 3.63 |
| L inf. aIC/extended amygdala | -28 | 8 | -24 | 405 | 5.17 |
| Cuneus | -18 | -78 | 0 | 35 | 3.58 |
| R cerebellum | 46 | -78 | -42 | 34 | 3.64 |
| L caudate nucleus | 16 | 14 | 12 | 48 | 3.82 |
| L thalamus | -16 | -8 | 8 | 29 | 3.58 |
| R hypothalamus | 8 | -12 | -8 | 30 | 3.64 |

Note: All regions are significant at *p* < 0.001, uncorrected for multiple comparisons with a minimum cluster size of 15 voxels. TRIs = task-related interferences, L = left, R = right, MPFC = medial prefrontal cortex, PHC = parahippocampal cortex, aIC = anterior insular cortex, Pre-SMA = pre-supplementary motor area.
