# Supplementary material for: Neural Correlates of Ongoing Conscious Experience: Both Task-Unrelatedness and Stimulus-Independence Are Related to Default Network Activity
Source: PLoS One. 2011 Feb 14;6(2):e16997. doi: 10.1371/journal.pone.0016997 (PMC3038939; doi:10.1371/journal.pone.0016997)
Supplement: Table S3 — Brain regions (outside a priori areas of interest) more active during task-related interferences compared to external distractions. (DOC) [file pone.0016997.s003.doc]

Table S3. Brain regions (outside a priori areas of interest) more active during task-related interferences compared to external distractions

|  | MNI coordinates | | |  |  |
| --- | --- | --- | --- | --- | --- |
|  | *x* | *y* | *z* | Voxels | *t* |
| R inferior parietal lobule | 48 | -58 | 48 | 372 | 4.16 |
| L post. inferior temporal gyrus | -62 | -52 | -26 | 18 | 3.50 |
| L inferior temporal gyrus | -46 | -16 | -32 | 33 | 3.48 |
| R middle frontal gyrus | 42 | 18 | 54 | 132 | 4.43 |
| L middle frontal gyrus | -36 | 24 | 58 | 27 | 3.48 |
| R orbital frontal gyrus | 44 | 40 | -26 | 22 | 3.60 |
| L orbital frontal gyrus | -46 | 26 | -24 | 106 | 4.15 |
| Pre-SMA | 4 | 40 | 62 | 187 | 4.30 |
| Cuneus | 6 | -104 | -4 | 61 | 3.84 |
| R cerebellum | 26 | -86 | -46 | 53 | 3.55 |

Note: All regions are significant at *p* < 0.001, uncorrected for multiple comparisons with a minimum cluster size of 15 voxels. L = left, R = right, Pre-SMA = pre-supplementary motor area.
