# Supplementary material for: Neural Correlates of Ongoing Conscious Experience: Both Task-Unrelatedness and Stimulus-Independence Are Related to Default Network Activity
Source: PLoS One. 2011 Feb 14;6(2):e16997. doi: 10.1371/journal.pone.0016997 (PMC3038939; doi:10.1371/journal.pone.0016997)
Supplement: Table S4 — Brain regions (outside a priori areas of interest) more active during mind-wandering compared to both task-related interferences and external distractions (conjunction analysis). (DOC) [file pone.0016997.s004.doc]

Table S4. Brain regions (outside a priori areas of interest) more active during mind-wandering compared to both task-related interferences and external distractions (conjunction analysis)

|  | MNI coordinates | | |  |  |
| --- | --- | --- | --- | --- | --- |
|  | *x* | *y* | *z* | Voxels | *t* |
| R dorsal MPFC | 8 | 48 | 16 | 21 | 3.38 |
| L anterior PHC | -24 | -10 | -38 | 93 | 4.04 |
| L inf. aIC/extended amygdala | -28 | 12 | -18 | 113 | 4.07 |
| L thalamus | -14 | -14 | -14 | 65 | 4.02 |

Note: All regions are significant at *p* < 0.001, uncorrected for multiple comparisons with a minimum cluster size of 15 voxels. L = left, R = right, MPFC = medial prefrontal cortex, PHC = parahippocampal cortex, aIC = anterior insular cortex.
